# Evidence of SARS-CoV-2 Transcriptional Activity in Cardiomyocytes of COVID-19 Patients without Clinical Signs of Cardiac Involvement

**DOI:** 10.3390/biomedicines8120626

**Published:** 2020-12-18

**Authors:** Gaetano Pietro Bulfamante, Gianluca Lorenzo Perrucci, Monica Falleni, Elena Sommariva, Delfina Tosi, Carla Martinelli, Paola Songia, Paolo Poggio, Stefano Carugo, Giulio Pompilio

**Affiliations:** 1 Unità di Anatomia Patologica, Dipartimento di Scienze della Salute, Università degli Studi di Milano, 20142 Milan, Italy; gaetano.bulfamante@unimi.it (G.P.B.); monica.falleni@unimi.it (M.F.); delfina.tosi@unimi.it (D.T.); carla.martinelli@unimi.it (C.M.); 2 Struttura Complessa di Anatomia Patologica e Genetica Medica, ASST Santi Paolo e Carlo, 20142 Milan, Italy; 3 Unità di Biologia Vascolare e Medicina Rigenerativa, Centro Cardiologico Monzino IRCCS, 20138 Milan, Italy; elena.sommariva@ccfm.it (E.S.); giulio.pompilio@ccfm.it (G.P.); 4 Unità per lo Studio delle Patologie Aortiche, Valvolari e Coronariche, Centro Cardiologico Monzino IRCCS, 20138 Milano, Italy; paola.songia@ccfm.it (P.S.); paolo.poggio@ccfm.it (P.P.); 5 Unità di Cardiologia, Dipartimento di Scienze della Salute, Università degli Studi di Milano, 20142 Milan, Italy; stefano.carugo@unimi.it; 6 Dipartimento di Scienze Cliniche e di Comunità, Università degli Studi di Milano, 20122 Milan, Italy

**Keywords:** COVID-19, SARS-CoV-2, heart, cardiomyocytes

## Abstract

Aims: A considerable proportion of patients affected by coronavirus respiratory disease (COVID-19) develop cardiac injury. The viral impact in cardiomyocytes deserves, however, further investigations, especially in asymptomatic patients. Methods: We investigated for SARS-CoV-2 presence and activity in heart tissues of six consecutive COVID-19 patients deceased from respiratory failure showing no signs of cardiac involvement and with no history of heart disease. Cardiac autopsy samples were collected within 2 h after death, and then analysed by digital PCR, Western blot, immunohistochemistry, immunofluorescence, RNAScope, and transmission electron microscopy assays. Results: The presence of SARS-CoV-2 into cardiomyocytes was invariably detected in all assays. A variable pattern of cardiomyocyte injury was observed, spanning from absence of cell death and subcellular alterations hallmarks, to intracellular oedema and sarcomere ruptures. In addition, we found active viral transcription in cardiomyocytes, by detecting both sense and antisense SARS-CoV-2 spike RNA. Conclusions: In this autopsy analysis of patients with no clinical signs of cardiac involvement, the presence of SARS-CoV-2 in cardiomyocytes has been detected, determining variable patterns of intracellular damage. These findings suggest the need for cardiologic surveillance in surviving COVID-19 patients not displaying a cardiac phenotype.

## 1. Introduction

The pandemic due to the infection of novel severe acute respiratory syndrome coronavirus (SARS-CoV-2) disease (COVID-19) [1] is still a burden worldwide. Besides SARS-CoV-2 virus lung tropism causing an atypical severe acute respiratory distress syndrome (ARDS) [2], evidence is documented about the involvement of several other organs, including the cardiovascular system. About 20–40% of hospitalized patients manifest a wide spectrum of symptoms related to heart injury, ranging from mild chest discomfort, palpitations, and arrhythmias to cardiogenic shock and fulminant heart failure [3]. Furthermore, myocardial injury has been documented in more than 50% of deceased patients [4] with up to 7% of COVID-19 related deaths being linked to myocarditis [5]. To date, it is not clearly understood whether the cause of the cardiovascular involvement of COVID-19 patients is due to SARS-CoV-2 direct cell damage or is rather secondary to the excessive immune system reaction. The systemic hyper-inflammatory status may act as a trigger for an aberrant host immune response mediated by natural killer (NK) cells, macrophages, and T-lymphocytes [6]. Inflammation-mediated leucocyte adhesion molecules on the endothelium may cause coronary dysfunction with ischemia [7] or acute coronary syndrome [8]. Alternatively, ARDS-induced hypoxia associated with systemic sepsis, fever, and hypotension may lead to myocardial injury [7,9] due to the oxygen supply and demand imbalance. On the other hand, SARS-CoV-2 has been found in vascular endothelial cells of patients affected by endotheliitis [10] and in cardiac macrophages of COVID-19 patients with cardiogenic shock and myocarditis [11,12]. Moreover, viral RNA has been previously reported in COVID-19 patients’ whole heart tissue [13,14], regardless of a cardiac phenotype [15,16]. The cell invasion mechanism of SARS-CoV-2 is mainly mediated by ACE2 receptor [17], which is highly expressed by several cardiac cell types, such as pericytes [18], cardiomyocytes [19], and endothelial cells [10,20]. Nonetheless, immunohistochemistry for viral antigen detection in cardiomyocytes has never been shown [21,22].

Here, we show, for the first time, multilayer data on the presence of SARS-CoV-2 sense and antisense RNA, viral proteins, and particles in cardiomyocytes of the autopsy heart samples of six patients deceased for COVID-19-dependent severe respiratory insufficiency in which no cardiac phenotype was manifest during hospitalization.

## 2. Materials and Methods

### 2.1. Data Availability

The raw data are available in Zenodo, at https://dx.doi.org/10.5281/zenodo.4303956.

### 2.2. Patients’ Autopsy Samples

Autopsies of 6 consecutive non selected patients involved in this study, 5 males and 1 female, aged 54–69 years (average age: 59.5 years) with a diagnosis of COVID-19, confirmed by CT analysis (Supplementary Figure S1) and polymerase chain reaction (PCR), were performed in a BSL3 equipped autopsy room at the Unit of Pathology of “ASST Santi Paolo e Carlo” of Milan. Examiners worn clad in all necessary DPIs. Examiners followed the recently published guidelines for performing autopsies and samples with suspected COVID-19 (SIAPEC-IAP) [23]. The autopsy examination was performed as soon as possible, by following the recently published recommendations. The period from death to post-mortem autopsy ranged from 1 to 2 h, documented instrumentally with a continuous and flat ECG for 20 min.

All patients had no clinical signs of left ventricle (LV) damage and died for SARS-CoV-2-dependent respiratory failure after hospitalization in the Intensive Care Unit. In Table 1 clinical characteristics of all 6 patients involved in this study have been summarized. Patients were in continuous observation for circulating values of reactive C-protein (RCP), fibrin degradation product D-dimer (D-DIMER), and ultrasensitive troponin I, but the values reported in Table 1 were the last acquired before death. The autopsy included complete histopathology and virology evaluation. Clinical records were checked for clinical conditions at hospitalization.

The data obtained from COVID-19-positive patients’ samples were compared with healthy control specimens, obtained from patients’ record of Unit of Pathology of “ASST Santi Paolo e Carlo” of Milan. The patient adopted as healthy control was a COVID-19-negative subject, dead for lung cancer, whose tissue specimens were collected in a period prior to the COVID-19 pandemic.

### 2.3. mRNA Extraction and Digital PCR Assay

Tissue samples for RNA were stored in RNAlater (Sigma-Aldrich, Saint Louis, MO, USA). Then, RNA was extracted from tissues by using TRIzol (ThermoFischer Scientific, Waltham, MA, USA) and Hybrid-R columns (GeneAll Biotechnology, Seoul, Korea), following the manufacturer’s instructions.

The RNA quantification was performed by spectrophotometer ND-1000 (NanoDrop^®^, EuroClone, Pero, Milan, Italy). For viral RNA quantification in lung and myocardial specimens, a one-step chip-based digital PCR was performed on a QuantStudio 3D Digital PCR System platform composed by the QuantStudio 3D Instrument, the Dual Flat Block GeneAmp PCR System 9700 and the QuantStudio 3D Digital PCR Chip Loader (ThermoFischer Scientific, Waltham, MA, USA). QuantStudio™ 3D Digital PCR Master Mix v2 (Applied Biosystems, Foster City, CA, USA) was used in combination with TaqMan primers (10006830, 10006831) and FAM probe (10006832) for viral N gene purchased from Integrated DNA Technologies (Integrated DNA Technologies, Coralville, IA, USA), the primers and probe for the N gene were controlled and authorized by the Centers for Disease Control and Prevention, and human RPLP0 gene (Hs99999902_m1) with a valve interstitial cell probe, as internal amplification control, purchased from Thermo Fisher Scientific. Reverse transcription (RT) was performed with the SuperScript III (Invitrogen, Carlsbad, CA, USA) following the manufacturer’s instructions.

PCR cycling consisted of initial denaturation at 96 °C for 10 min followed by denaturation step at 98 °C for 30 s and annealing/extension at 56 °C for 1 min (40 cycles), and a final extension at 60 °C for 5 min. Analyses were executed with the online version of the QuantStudio 3D AnalysisSuite (ThermoFischer Scientific Cloud, Waltham, MA, USA) following the manufacturer’s instructions.

### 2.4. Western Blot Analysis

Heart and lung tissues were fragmented by Spectrum™ Bessman Tissue Pulverizers (Fisher Scientific, Hampton, NH, USA) and lysed in cell lysis buffer (Cell Signaling Technology, Danvers, MA, USA) supplemented with protease and phosphatase inhibitor cocktails (Sigma-Aldrich, Saint Louis, MO, USA).

After quantification by BCA Protein Assay (ThermoFischer Scientific, Waltham, MA, USA), 30 μg of total protein extracts (both for heart and lung) were subjected to SDS-PAGE and transferred onto a nitrocellulose membrane. The membranes were blocked for 1 h at room temperature (RT) in Wash Buffer (Tris Buffer Sulfate 1×, 0.1% Tween 20) supplemented with 5% Bovine Serum Albumine (BSA) and then incubated over-night (O/N) at 4 °C with the appropriate primary antibody. Primary antibodies adopted for western blot analysis were specific for ACE2 (R&D System, Minneapolis, MN, USA), SARS-CoV-2 nucleoprotein (Sino Biological, Düsseldorfer, Germany), SARS-CoV-2 spike protein (GeneTex, Irvine, CA, USA), and GAPDH (Santa Cruz Biotechnology, Dallas, TX, USA).

The membranes were incubated with peroxidase-conjugated secondary antibodies (GE Healthcare, Chicago, IL, USA) for 1 h. Signals were visualized using the Western blotting chemiluminescence substrate LiteUP (EuroClone, Pero, Milan, Italy). Images were acquired with the ChemiDoc system (Bio-Rad Laboratories, Hercules, CA, USA).

### 2.5. Immunostainings and Histochemistry

Heart and lung samples were formalin-fixed immediately after autopsy. Heart samples were mapped as follows: full thickness samples from ventricles, atri (2 for each side), and the interventricular septum (2). Lung samples were mapped as follows: 4 samples, 2 from anterior and 2 from posterior—2 cranial and 2 caudal for each lobe; areas of specific macroscopical interest were also sampled. Specimens were routinely fixed in 10% buffered formaldehyde for 48 h, as recommended [23], and then 3 µm sections were processed for histologic evaluation and for immunostainings.

Immunohistochemistry was performed with the automatic immunostainer DAKO OMNIS (DAKO, Glostrup, Denmark), using staining kit with magenta and brown chromogens, routinely applied in our laboratory. The magenta staining was adopted to avoid misinterpretation with lipofuscin granularity typical of cardiomyocyte cytoplasm.

The histochemistry with phosphotungstic acid haematoxylin (PTAH) was performed according to manufacturer’s protocol (BioOptica, Milan, Italy).

Primary antibodies adopted for immunohistochemistry and immunofluorescence were specific for SARS-CoV-2 nucleoprotein (Cat#40143-R019, Sino Biological, Düsseldorf, Germany), SARS-CoV-2 spike protein (clone: 1A9, GeneTex, Irvine, CA, USA), sarcomeric α-actin (α-SARC, clone: EA-53, AbCam, Cambridge, UK), and cytokeratin 7 (CK7, clone: OV-TL12/30, Agilent Dako, Santa Clara, CA, USA).

As for SARS-CoV-2 nucleoprotein, samples from lungs of patients under study, with immunoreactivity in reactive pneumocytes, macrophages and epithelial respiratory cells were used as controls. Negative controls were also included in the study by omitting the primary antibody. For all the stainings, archival heart and lung tissue samples from patients dead for diseases other than COVID-19 and cardiologic causes were used as controls (Healthy Control).

Immunofluorescence images and analyses were performed by using a Zeiss LSM710 confocal microscope (Carl Zeiss, Oberkochen, Germany).

Except for immunofluorescence, all slides were digitalized with the NanoZoomer S360 Digital slide scanner C13220-01 (Hamamatsu Photonics, Milan, Italy).

### 2.6. RNAscope Assays

The RNAScope assays (ACD, Bio-technè, Minneapolis, MN, USA) were performed on 3 µm formalin-fixed, paraffin-embedded sections, following Wang et al. published method [24], by using specific probes for sense and antisense SARS-CoV-2 RNA spike sequences. Nuclei were counterstained with haematoxylin and the images were acquired by AxioImager microscope (Carl Zeiss, Oberkochen, Germany).

### 2.7. TUNEL Assay

Nuclei with fragmented DNA, associated with necrosis and/or apoptosis, were visualized by using a terminal deoxyribonucleotidyl transferase-mediated dUTP-digoxigenin nick end labeling (TUNEL) detection kit (Roche, Mannheim, Germany). At last, nuclei were counterstained with Hoechst (ThermoFischer Scientific, Waltham, MA, USA) and images were acquired by ApoTomeII microscope (Carl Zeiss, Oberkochen, Germany).

### 2.8. Transmission Electron Microscopy

Cardiac tissue samples for ultrastructural analysis were fixed in 2.5% glutaraldehyde in 0.13 M phosphate buffer pH 7.2–7.4 for 2 h, post-fixed in 1% osmium tetroxide, dehydrated through graded ethanol and propylen oxide, and embedded in epoxy resin. Ultrathin sections of 50 to 60 nm not counterstained were observed with transmission electron microscope Leo 912ab (Carl Zeiss, Oberkochen, Germany).

## 3. Results

### 3.1. Patients’ Clinical and Autoptic Features

The patient features reported in Table 1 highlight that all the subjects died from respiratory failure. Supplementary Figure S1 shows a representative result of a pulmonary computerized tomography (CT), pathognomonic for COVID-19 disease. Only 1 out of 6 presented a slightly altered cardiac clinical profile, due to a mild hypertrophic left ventricle (LV), likely secondary to hypertension, occurring before the hospitalization for COVID-19 disease. Importantly, all patients did not show alterations of ultrasensitive Troponin I; 2 out of 6 were hypertensive; 2 out of 6 showed hypercholesterolemia, none were diabetic, and one presented thyroid dysfunction. All six patients were assisted by mechanical ventilation in the intensive care unit for a period ranging from 6 to 21 days. Concerning autopsy details, hearts ranged in size from 410 to 750 g (normal: 365 ± 71 g), with cardiomegaly related to right ventricle dilation, due to lung overload. On cut surface, cardiac tissue was firm, brown, free of lesions, with some delicate areas of myocardial sclerosis in 1 out of 6 patient LVs; the coronary system was unremarkable, with no significant stenosis nor thrombosis.

### 3.2. SARS-CoV-2 Genome and Proteins Were Present in Cardiac Samples of COVID-19 Patients

**Figure 1:**
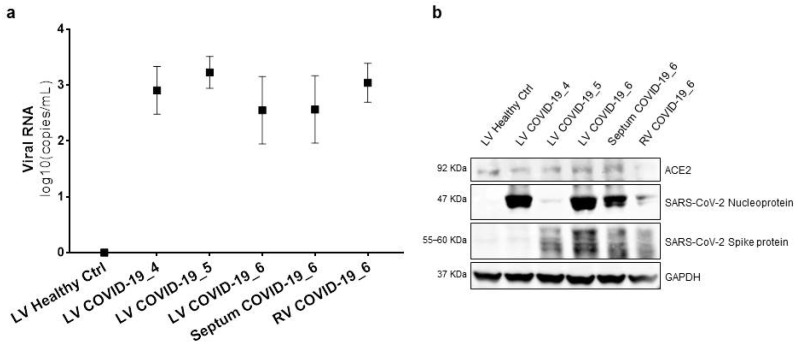
RNA and protein of SARS-CoV-2 were present in cardiac samples of COVID-19 patients. (**a**) Chip-based digital PCR for SARS-CoV-2 N sequence in total RNA extracts from tissues of 3 out of 6 different patients and different cardiac regions. Raw expression values are expressed as log10 (copies/mL). The results are expressed as median and confidence interval (CI 95%). (**b**) Western blot analysis for ACE2, SARS-CoV-2 nucleoprotein, and spike protein in total protein extracts from tissues of 3 out of 6 different patients and different cardiac regions. GAPDH has been adopted as loading control. LV, left ventricle; Septum, interventricular septum; RV, right ventricle.

We initially checked SARS-CoV-2 presence in lung extracts of patients involved in this study by chip-based digital PCR and Western blot analyses (Supplementary Figure S2). After that, we confirmed SARS-CoV-2 cardiac tropism in these patients, by examining the presence of viral RNA in total RNA extracts from heart tissue samples with the same PCR technique. Viral RNA was found in all COVID-19 heart specimens, but not in the healthy control. The number of copies of SARS-CoV-2 RNA molecules ranged from 4.44 to 5.33 log10 (copies/mL) (Figure 1a).

The Western blot analysis performed on total protein cardiac autopsy specimen lysates confirmed that the well-known viral gate of host cells’ ACE2 receptor [17] was expressed in the cardiac tissue [20]. Similar levels of expression were found in different cardiac districts, and of note, independently of COVID-19. Furthermore, we showed for the first time that SARS-CoV-2 viral proteins are detectable in COVID-19 patient hearts, and no traces were found in the healthy controls. In detail, nucleoprotein and spike protein were expressed at different levels in the analysed patients (Figure 1b), raising the hypothesis that the viral genome is actively transcribed into cardiomyocytes.

### 3.3. SARS-CoV-2 Is Localized in Cardiomyocytes of COVID-19 Patients in Terms of Viral Proteins and RNA

**Figure 2:**
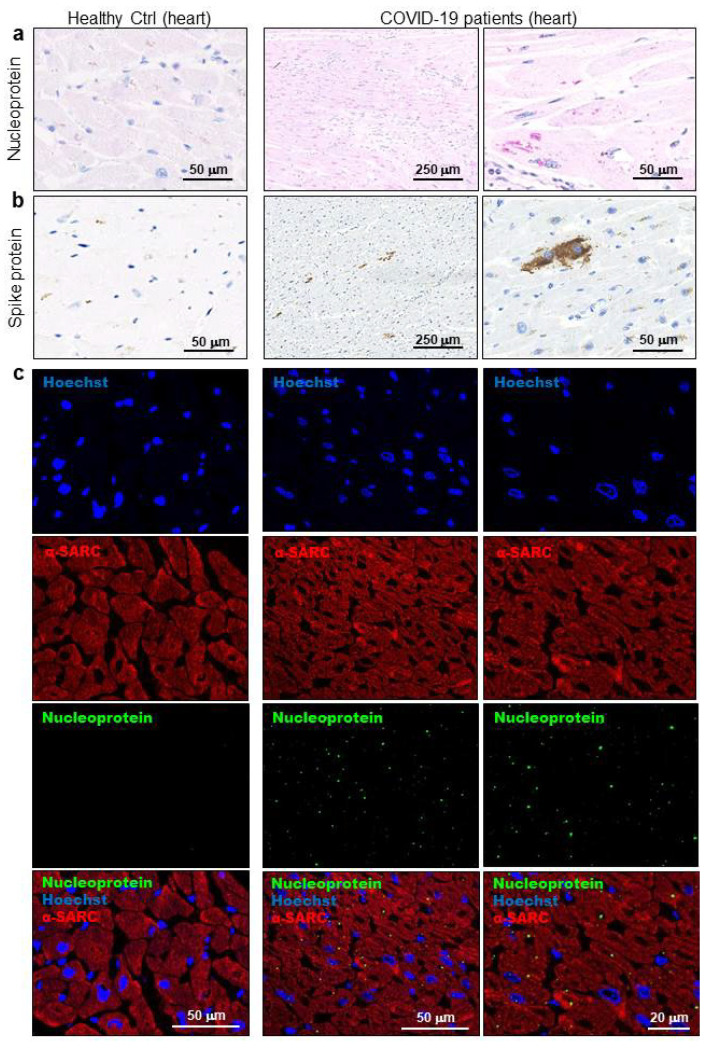
SARS-CoV-2 proteins were detectable in cardiomyocytes of COVID-19 patients. (**a**,**b**) Representative images of immunohistochemistry assays on 3 μm slides of formalin-fixed, paraffin-embedded left ventricle specimens from healthy controls (Healthy Ctrl, left panels) and COVID-19 patients (central and right panels) for SARS-CoV-2 nucleoprotein ((**a**), in red) and spike protein ((**b**), in brown). (**c**) Representative images of immunofluorescence assay on 3 μm slides of formalin-fixed, paraffin-embedded left ventricle specimens from healthy controls (Healthy Ctrl, left panels) and COVID-19 patients (central and right panels) for SARS-CoV-2 nucleoprotein (green) and sarcomeric α-actin (α-SARC, red). Nuclei have been stained by Hoechst (blue). Magnification as per scale bars.

The light microscopy analyses performed firstly allowed us to define all COVID-19 cases as similar by microscopic aspects. In detail, the immunohistochemical analyses confirmed positivity for COVID-19 not only in the lungs (Supplementary Figure S3) but also in LV cardiac samples for SARS-CoV-2 nucleoprotein and spike protein (Figure 2a,b). Of note, viral proteins were found within cardiomyocytes cell body, and more specifically, in cytosolic areas of lipofuscin (Supplementary Figure S4a). This finding was confirmed by immunofluorescence co-staining of viral nucleoprotein with sarcomeric α-actin (αSARC)-positive cells (Figure 2c).

**Figure 3:**
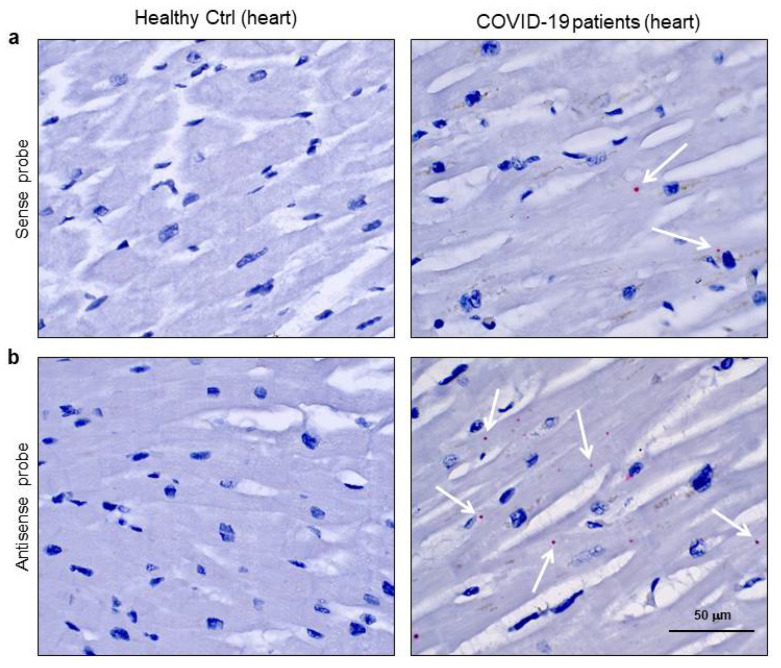
SARS-CoV-2 sense and antisense RNA were localized in cardiomyocytes of COVID-19 patients. (**a**,**b**) Representative images of spatially resolved viral RNA detection by RNAScope assay on 3 μm slides of formalin-fixed, paraffin-embedded left ventricle specimens from healthy control (Healthy Ctrl, left panels) and COVID-19 patients (right panels) for SARS-CoV-2 sense (**a**) and antisense (**b**) probes for spike protein RNA sequences. Fast Red dots indicate viral RNA presence (white arrows). Nuclei have been counterstained with haematoxylin. Magnification as per scale bars.

In order to determine the presence of SARS-CoV-2 as transcriptionally active virus in cardiomyocytes, we performed the RNAScope assay (Figure 3) [24]. In detail, this in situ hybridization was performed by separately using two different probes, one recognising the sense and the other the antisense spike protein RNA. The use of both probes allowed the distinction of viral RNA genome from its transcript. Firstly, we checked the technique’s effectiveness by probing COVID-19 lung specimens, as shown in the Supplementary Figure S5. Then, we tested the cardiac samples of the same COVID-19 patients for both sense (Figure 3a) and antisense (Figure 3b) probes. Signals were obtained with both probes in the cardiomyocytes of COVID-19 patients only, but the most abundant staining resulted from the probe detecting the actively transcribed virus (Figure 3b). The images for positive and negative control probes on heart samples are shown in Supplementary Figure S6.

### 3.4. Cardiomyocytes Containing SARS-CoV-2 Do Not Show Cell Death Signs, But Display Intracellular Alterations

**Figure 4:**
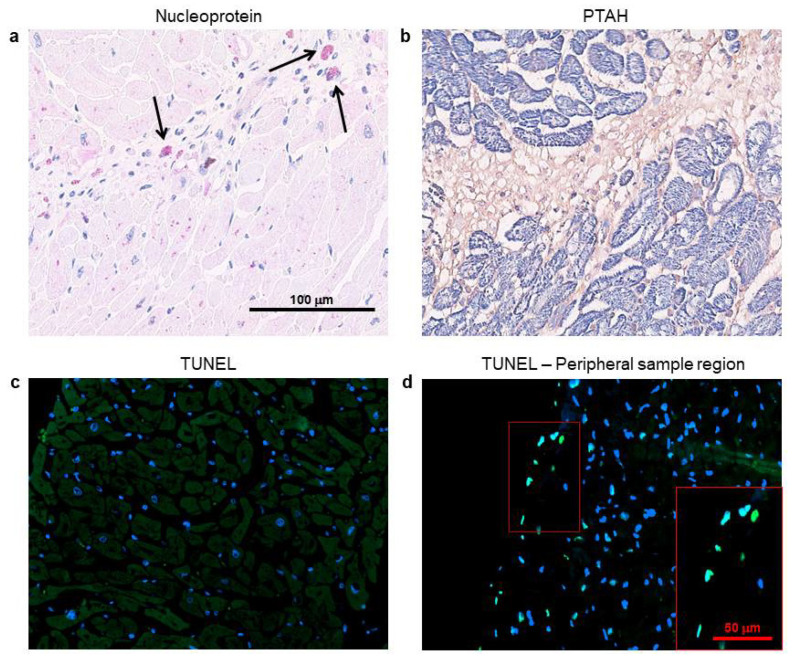
Several cardiomyocytes containing SARS-CoV-2 nucleoprotein display intracellular alterations, but not cell death signs. (**a**,**b**) Representative images on consecutive slides of immunohistochemistry assay for SARS-CoV-2 nucleoprotein (**a**) and PTAH staining ((**b**), sarcomere structures in violet/blue), performed on left ventricle specimens from COVID-19 patients. Black arrows indicate strongly immunoreactive interstitial cells for SARS-CoV-2 nucleoprotein (in red), with a morphology consistent with macrophages. (**c**,**d**) Representative images of TUNEL assay on 3 μm slides of formalin-fixed, paraffin-embedded left ventricle specimens from COVID-19 patients. Damaged DNA is marked in green; nuclei have been counterstained with Hoechst (blue). Magnification as per scale bars.

In Figure 4, the immunohistochemistry for SARS-CoV-2 nucleoprotein and PTAH staining, performed on consecutive heart sections, highlighted several microscopic scenarios. Firstly, we confirmed literature data on SARS-CoV-2-positivity in cardiac interstitial cells (Figure 4a, black arrows) [11,12]. Then, we observed that not all the cardiomyocytes contain the virus, and among those positive for the viral nucleoprotein, we found living and unaltered cells (Figure 4a). Nonetheless, we appreciated, through phosphotungstic acid haematoxylin (PTAH) staining, isolated or grouped cardiomyocytes showing atypical degenerative aspects, such as increased cell volumes with intracellular oedema, together with rarefied, disarranged, disrupted, and plurifocally clumped myofibrils (Figure 4b).

In order to investigate whether these interesting features, concerning cardiac cell alterations, may be linked to apoptotic/necrotic processes activation, we performed TUNEL assays on all the subject samples and we detected no cell death signs, except in the peripheral sample regions (Figure 4c,d).

Vascular modifications were unremarkable; no endothelial viral cytopathic effects nor endotheliitis were documented. Furthermore, a low grade of inflammatory infiltrates was observed in cardiac tissue, especially in sub-pericardiac regions without a specific distribution (Supplementary Figure S4b). Among cardiac interstitial cells, we displayed sporadic amount of interstitial macrophages with strong immunoreactivity for SARS-CoV-2 nucleoprotein (Supplementary Figure S4c).

**Figure 5:**
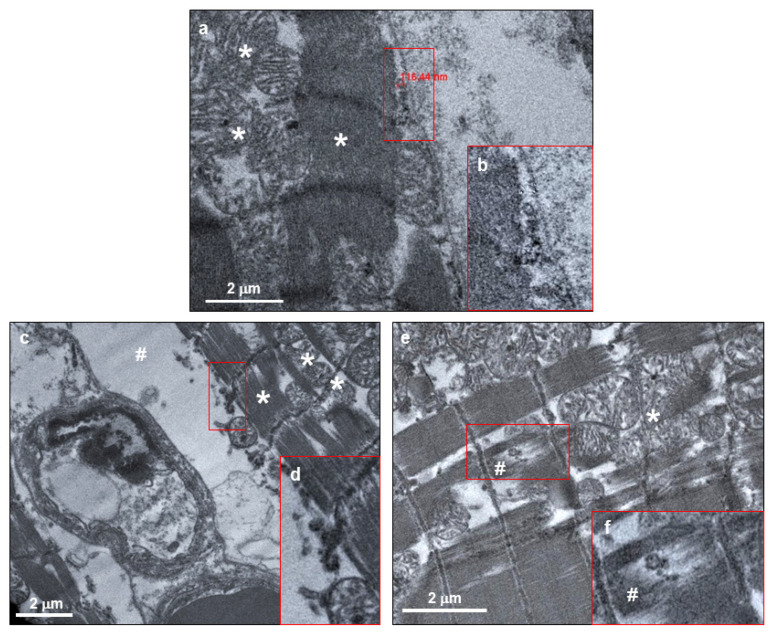
SARS-CoV-2 virions were localized within both normal and altered cardiomyocytes of COVID-19 patients. (**a**,**c**,**e**) Electron micrograph showing cardiomyocytes containing viral particles, compatible with crown-shaped SARS-CoV-2. The viral particle diameter (**a**) is about 116 nm. (**b**,**d**,**f**) High magnifications of red squared areas of panels a, c, and e, respectively. In all panels, asterisks indicate normal mitochondrial and sarcomere structures, while cytoplasmic and sarcomere alterations are indicated with hashes.

The investigation by electron microscopy confirmed the previously obtained evidence on the SARS-CoV-2 presence in cardiomyocytes. Indeed, single and/or grouped spherical particles about 130 nm in diameter, with electron dense periphery characterized by crown-shaped small external projections and a morphology compatible with SARS-CoV-2 were clearly visible in the intracellular space between sarcomeres and sarcolemma of cardiomyocytes (Figure 5a,b, arrows).

As for immunohistochemistry and histology assays, the cardiomyocyte phenotype is not univocal: while some cardiomyocytes hosting SARS-CoV-2 did not present any evident abnormality in terms of cellular structures, other cardiac cells presented peculiar degenerative features, such as excessive intracellular area between sarcolemma and sarcomere structure (Figure 5c,d) and abnormal, disrupted, and clumped myofibrils (Figure 5e,f). Regarding the former alteration, this cardiomyocyte swelling (Figure 5c, hashtag) may microscopically explain the cardiac oedema, previously observed in histological examinations. Noteworthily, the sarcomere rupture occurs in co-localization of SARS-CoV-2 viral particle (Figure 5f, hashtag).

Although the microscopy investigation was compatible with cardiomyocyte damage, it is important to highlight that, at the ultrastructural level, the sarcolemma and mitochondria were unaltered and structurally undamaged (Figure 5a,c,e, asterisks), thereby not being suggestive of cardiomyocyte death.

## 4. Discussion

In this work, we reported the presence of SARS-CoV-2 in cardiomyocytes of a series of six autoptic samples, obtained from patients deceased by respiratory failure showing no clinical signs of cardiac involvement and no previous history of heart disease. The novelty of our study consists of the observation into cardiomyocytes of (i) SARS-CoV-2 RNA and protein localization and (ii) active viral transcription of both sense and antisense RNA for SARS-CoV-2 spike protein. 

Although several papers have already reported the presence of SARS-CoV-2 RNA in the heart [12,15,16], none of them have detected viral localization in cardiomyocytes. We here demonstrated, by means of several techniques, the undisputable presence of active SARS-CoV-2 in COVID-19 patients’ cardiomyocytes, in terms of viral transcript detection (by digital PCR and RNAScope assays), viral protein detection (by Western blot, immunohistochemistry, and immunofluorescence), and full virions identification (by electron microscopy). Specifically, a clear signal for the SARS-CoV-2 spike protein was found in cardiomyocytes only; granular diffuse immunostaining of the SARS-CoV-2 nucleoprotein antigen showed its presence both in cardiomyocytes and in interstitial inflammatory cells, mostly in macrophages. These latter data are in line with viral particle detection in cardiac CD68^+^ cells described by Tavazzi et al. [11] and with RNA viral presence in macrophages recently reported by Lindner and colleagues [12], and suggest a role of macrophages as preferential SARS-CoV-2 carriers from lungs throughout the other organ tissues. On the contrary, the detection of viral protein and transcriptional activity adds unprecedented insights about SARS-CoV-2 protein expression and localization in the cardiac muscle. These findings are even more relevant considering that heart samples were taken from patients deceased for COVID-19 with no clinical signs of cardiac involvement.

Importantly, in this regard, we have observed in cardiomyocytes harbouring SARS-CoV-2 different patterns of intracellular alterations. To date, even if studies based on endomyocardial biopsies or isolated autopsy description previously revealed cardiomyocyte alterations in COVID-19 patients’ hearts [25], no clear evidence of a concomitant viral infection was provided. Here we demonstrated, for the first time, cardiac cell damage in the presence of immunostaining for SARS-CoV-2 [11,12,13,20]. Scattered and focal degenerative cardiomyocyte features were further confirmed by electron microscopy image analyses at the ultrastructural level. Several cardiomyocytes showed an altered cell structure, with increased cell area and intracellular oedema, suggesting cardiomyocyte swelling. Moreover, damaged sarcomeres, with disrupted and clumped myofilaments, were also observed, in some cases associated with rarefaction of myofibrils. It is important to point out that none of these cells showed disrupted or damaged sarcolemma. As for cardiac cell swelling, this microscopic event may be interpreted as a precocious subclinical sign of cell damage, potentially leading to myocardial interstitial oedema formation [26]. Moreover, focal myofibrillar lysis is in line with observation previously described by Tavazzi et al. [11] and might correspond with the atypical degenerative characteristics reported in myocytes from COVID-19 patient autoptic tissues [25]. The small number of virions we have observed is in line with the low SARS-CoV-2 loads, previously reported by Escher et al. [14] in a series of endomyocardial biopsies from patients with an acute cardiac phenotype and with the reported absence of immunoreactivity for SARS-CoV-2 nucleocapsid protein, described by Adachi et al. in a cardiac autopsy case report [22].

Noteworthily, available histological findings of SARS-CoV-2 detection in the heart tissue reported in the literature often lack a correlation with clinical data (e.g., cardiac troponin levels). Therefore, the relevance and the incidence of COVID-19 cardiac involvement still remain unclear with respect to an overt clinical cardiologic phenotype. Our data suggest that even the cardiomyocyte compartment may be infected by the SARS-CoV-2 virus, and may present microscopic cardiomyocyte alterations; however, this does not necessarily lead to clinical grade cardiac damage. Furthermore, the presence of viral nucleoprotein signal in cardiomyocytes and in lipofuscin granules may further suggest an attempt of functional cardiac cells to fight viral infection.

## 5. Conclusions

This study may be relevant for COVID-19 patients’ follow-ups, and specifically suggest the need to redefine post-discharge cardiologic surveillance guidelines for surviving COVID-19 patients, even in the absence of overt cardiac phenotype, since the cardiovascular long-term outcome of COVID-19 patients may mirror SARS patients, who developed cardiovascular abnormalities in 40% of recovered cases in a 12-year-long follow-up period [5].

## 6. Limitations of the Study

This study has limitations inherent to the number of patients’ autopsies included. Moreover, in one out of six patients only an echocardiography examination was performed, due to the severity of clinical presentation and the need for recovery in the emergency department. Nevertheless, the lack of alterations of ultrasensitive troponin I in all patients included in this study is consistent with the absence of overt cardiac involvement. We deliberately did show any statistical analysis, since a complete absence of any SARS-CoV-2 signs in healthy control samples was observed. Obviously, further studies will be necessary to elucidate the relationships between SARS-CoV-2 infection and macroscopic cardiac involvement leading to heart damage.

## Figures and Tables

**Table 1:** Clinical data of the patients analysed.

Patient	1	2	3	4	5	6
Sex/Age	M/59	M/54	M/56	M/69	M/55	F/64
Cause of death	Respiratory failure	Respiratory failure	Respiratory failure	Respiratory failure	Respiratory failure	Respiratory failure
Previously myocardial damages	//	//	//	//	//	Mildly hypertrophic LV. EF > 50%
RCP (mg/L)	134.4	67.7	118.1	53.8	82.8	135
D-DIMER (ng/mL)	2242	507	783	583	2987	906
TROPONIN I ultrasensitive (ng/mL)	<0.012	0.022	<0.012	<0.012	<0.012	<0.012
Hypertension	YES	NO	NO	NO	NO	YES
Hypercholesterolemia	YES	NO	NO	YES	NO	NO
Diabetes	NO	NO	NO	NO	NO	NO
Thyroid dysfunction	NO	NO	NO	NO	NO	YES
SARS-CoV-2 swab	Positive at admission	Positive at admission	Positive at admission	Positive at admission	Positive at second test	Positive at admission
Pulmonary outcome	Bilateral interstitial pneumonia due to COVID-19	Bilateral interstitial pneumonia due to COVID-19	Bilateral interstitial pneumonia due to COVID-19	Bilateral interstitial pneumonia due to COVID-19	Bilateral interstitial pneumonia due to COVID-19 and *E.Cloacae*	Bilateral interstitial pneumonia due to COVID-19
Hospitalization length (days)	16	29	27	38	18	7
Oxygen saturation (%)	79.7	92.2	92.2	94.1	87.0	90.5
Type of ventilation	NIV/CPAP	CPAP/MV	NIV/MV	NIV/MV	NIV/CPAP/MV	NIV/CPAP/MV
Pronation cycles	Multiple	Multiple	Multiple	Multiple	Multiple	2

Legend: // = not reported; LV = left ventricle; EF = ejection fraction; RCP = reactive c-protein; NIV = non-invasive ventilation; CPAP = continuous positive airway pressure; MV = mechanical ventilation.

## Supplementary Materials

The following are available online at https://www.mdpi.com/2227-9059/8/12/626/s1. Figure S1: Representative Computed Tomography image of a COVID-19 patient. Figure S2: RNA and protein of SARS-CoV-2 are present in lung samples of COVID-19 patients. Figure S3: SARS-CoV-2 proteins are detectable in lung tissue of COVID-19 patients. Figure S4: SARS-CoV-2 nucleoprotein localization in lipofuscin granule area of cardiomyocytes and in macrophages, and inflammatory infiltrates of heart samples of COVID-19 patients. Figure S5: SARS-CoV-2 sense and antisense RNA are localized in the lung specimens of COVID-19 patients. Figure S6: RNAScope positive and negative controls on heart samples from healthy subject and COVID-19 patients.
