# Supplementary material for: Evidence of SARS-CoV-2 Transcriptional Activity in Cardiomyocytes of COVID-19 Patients without Clinical Signs of Cardiac Involvement
Source: Biomedicines. 2020 Dec 18;8(12):626. doi: 10.3390/biomedicines8120626 (PMC7767122; doi:10.3390/biomedicines8120626)
Supplement: Supplementary file 1 [file biomedicines-08-00626-s001.pdf]

Evidence of SARS-CoV-2 transcriptional activity in cardiomyocytes of COVID-19 patients without clinical signs of cardiac involvement

Gaetano Pietro Bulfamante[1,2*], Gianluca Lorenzo Perrucci[3*✉], Monica Falleni[1,2], Elena Sommariva[3], Delfina Tosi[1], Carla Martinelli[1], Paola Songia[4], Paolo Poggio[4], Stefano Carugo[5], and Giulio Pompilio[3,6]

[1] Unità di Anatomia Patologica, Dipartimento di Scienze della Salute, Università degli Studi di Milano, Milan, Italy

[2] Struttura Complessa di Anatomia Patologica e Genetica Medica, ASST Santi Paolo e Carlo, Milan, Italy

[3] Unità di Biologia Vascolare e Medicina Rigenerativa, Centro Cardiologico Monzino IRCCS, Milan, Italy

[4] Unità per lo Studio delle Patologie Aortiche, Valvolari e Coronariche, Centro Cardiologico Monzino IRCCS, Milano, Italy

[5] Unità di Cardiologia, Dipartimento di Scienze della Salute, Università degli Studi di Milano, Milan, Italy

[6] Dipartimento di Scienze Cliniche e di Comunità, Università degli Studi di Milano, Milan, Italy

[*] *equal contribution as first author*

Short title: SARS-CoV-2 in cardiomyocytes of COVID-19 patients

✉ **Corresponding author**

Gianluca Lorenzo Perrucci, PhD (ORCID: 0000-0002-4758-6040)
Unità di Biologia Vascolare e Medicina Rigenerativa
Centro Cardiologico Monzino-IRCCS
Via privata Carlo Parea, 4 - 20138 Milan, Italy
e-mail: gianluca.perrucci@ccfm.it
Tel.: +390258002754
Fax: +390258002342

Supplementary Figures

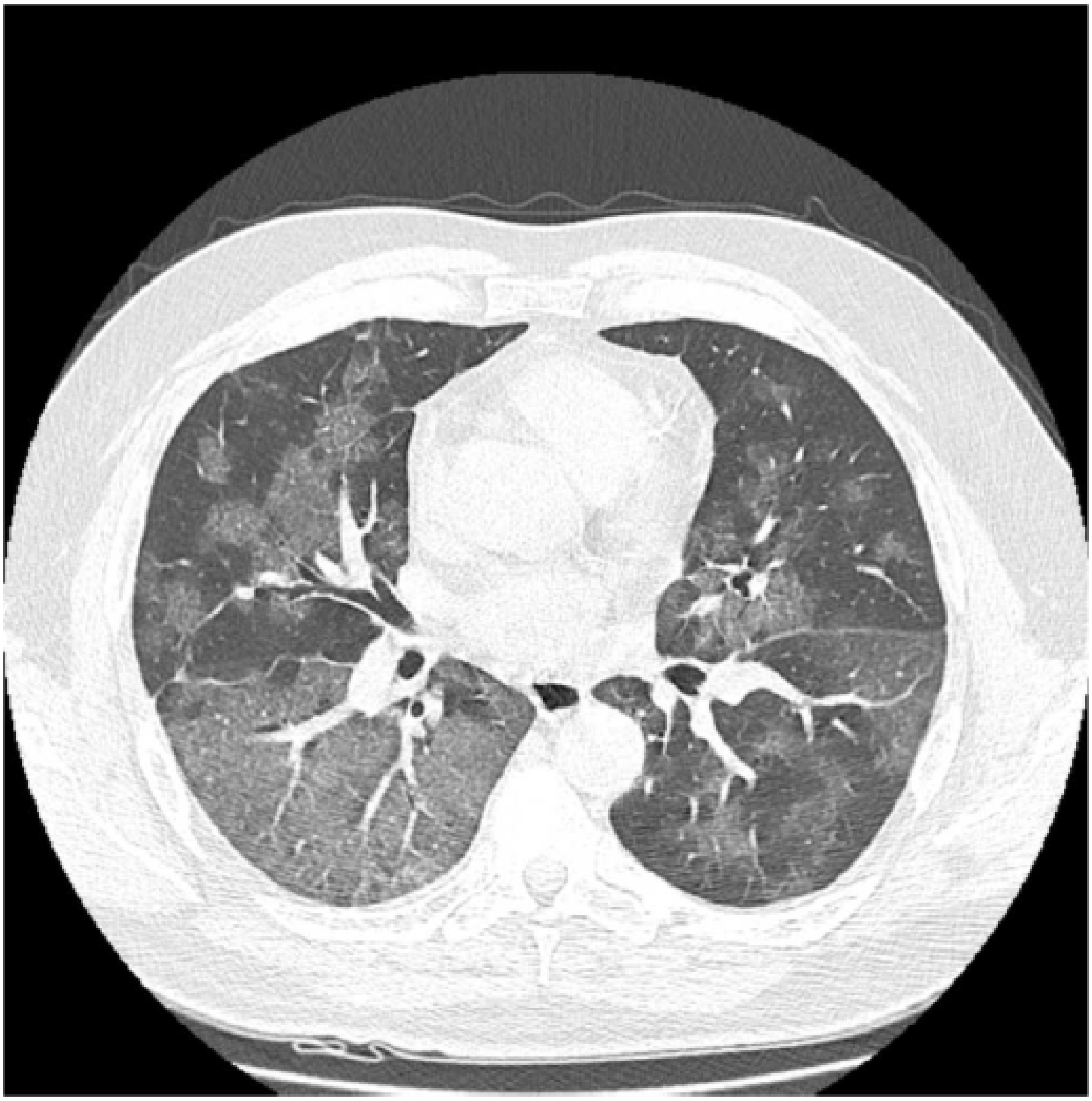

Supplementary Figure 1 – Representative Computed Tomography image of a COVID-19 patient. Bilateral pathognomonic lung lesion patterns typical of COVID-19 pneumonia are visible.

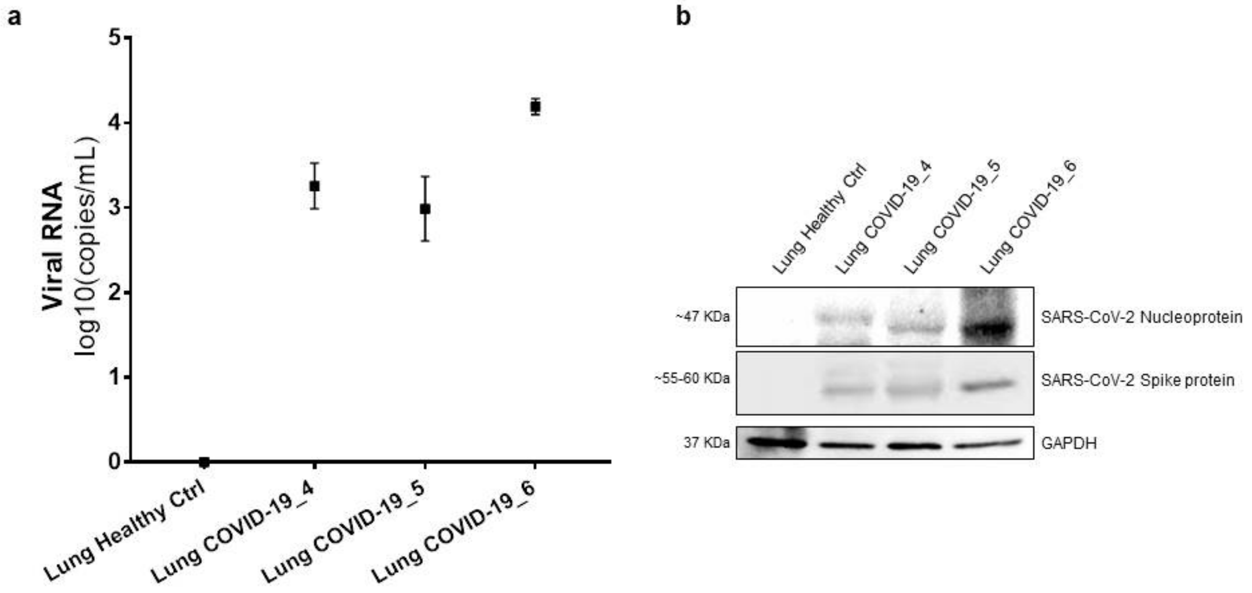

Supplementary Figure 2 - RNA and protein of SARS-CoV-2 are present in lung samples of COVID-19 patients. a) Chip-based digital PCR for SARS-CoV-2 N sequence in total RNA extracts from lung tissues of 3 out of 6 different patients. Raw expression values were expressed as log10(copies/mL). The results are expressed as median and confidence interval (CI 95%). **b)** Western blot analysis for SARS-CoV-2 nucleoprotein and spike protein on total protein extracts from lung tissues of 3 out of 6 different patients. GAPDH has been adopted as loading control.

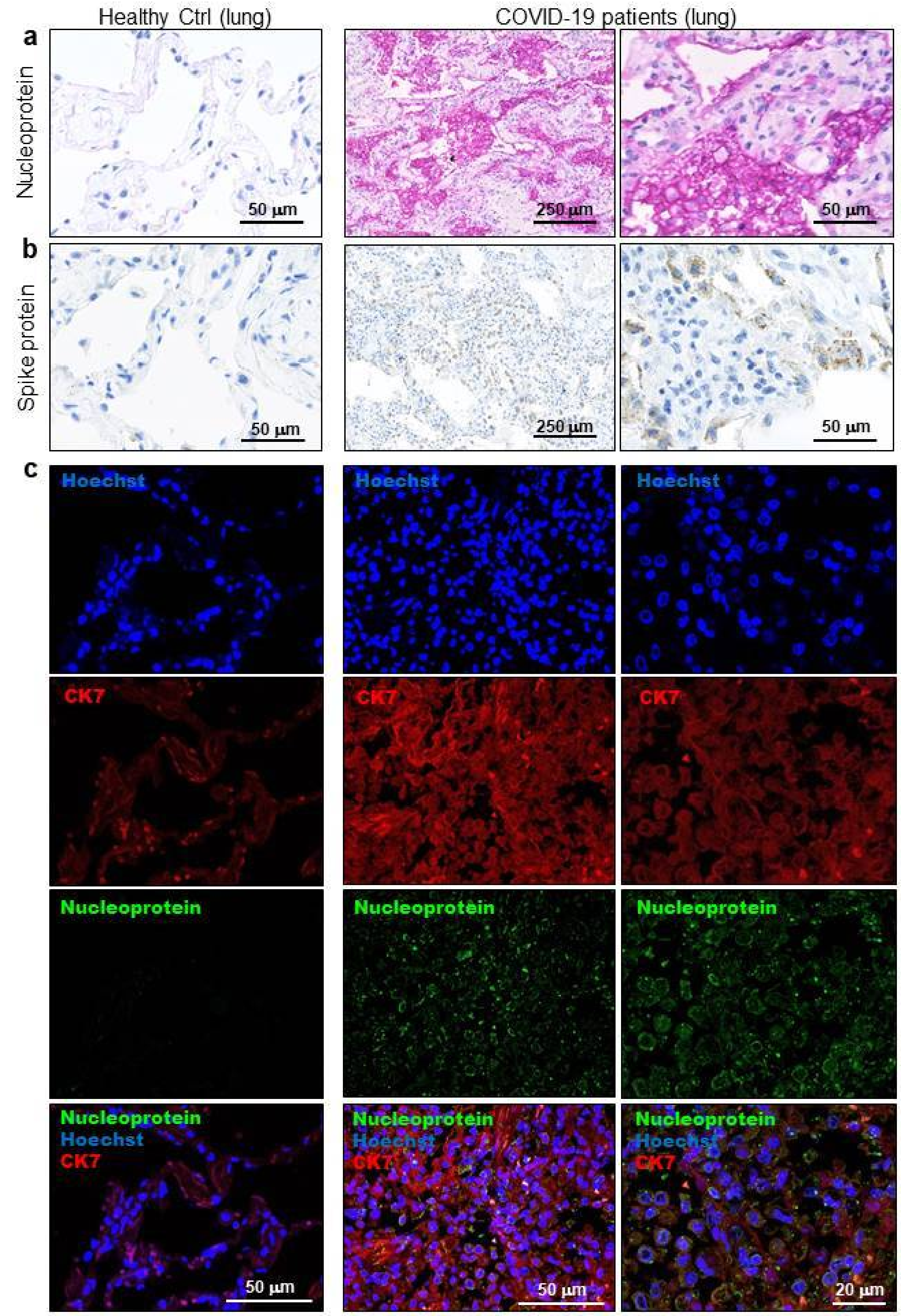

Supplementary Figure 3 - SARS-CoV-2 proteins are detectable in lung tissue of COVID-19 patients. a-b) Representative images of immunohistochemistry assays on 3µm-slides of formalin-fixed paraffin-embedded lung specimens from healthy control (Healthy Ctrl, left panels) and COVID-19 patients (COVID-19, central and right panels) for SARS-CoV-2 nucleoprotein (**a**, in red) and spike protein (**b**, in brown). **c)** Representative images of immunofluorescence assay on 3µm-slides of formalin-fixed paraffin-embedded lung specimens from healthy control (Healthy Ctrl, left panels) and COVID-19 patients (COVID-19, central and right panels) for SARS-CoV-2 nucleoprotein (green) and cytokeratin 7 (CK7, red). Nuclei have been stained by Hoechst (blue). Magnification as scale bars.

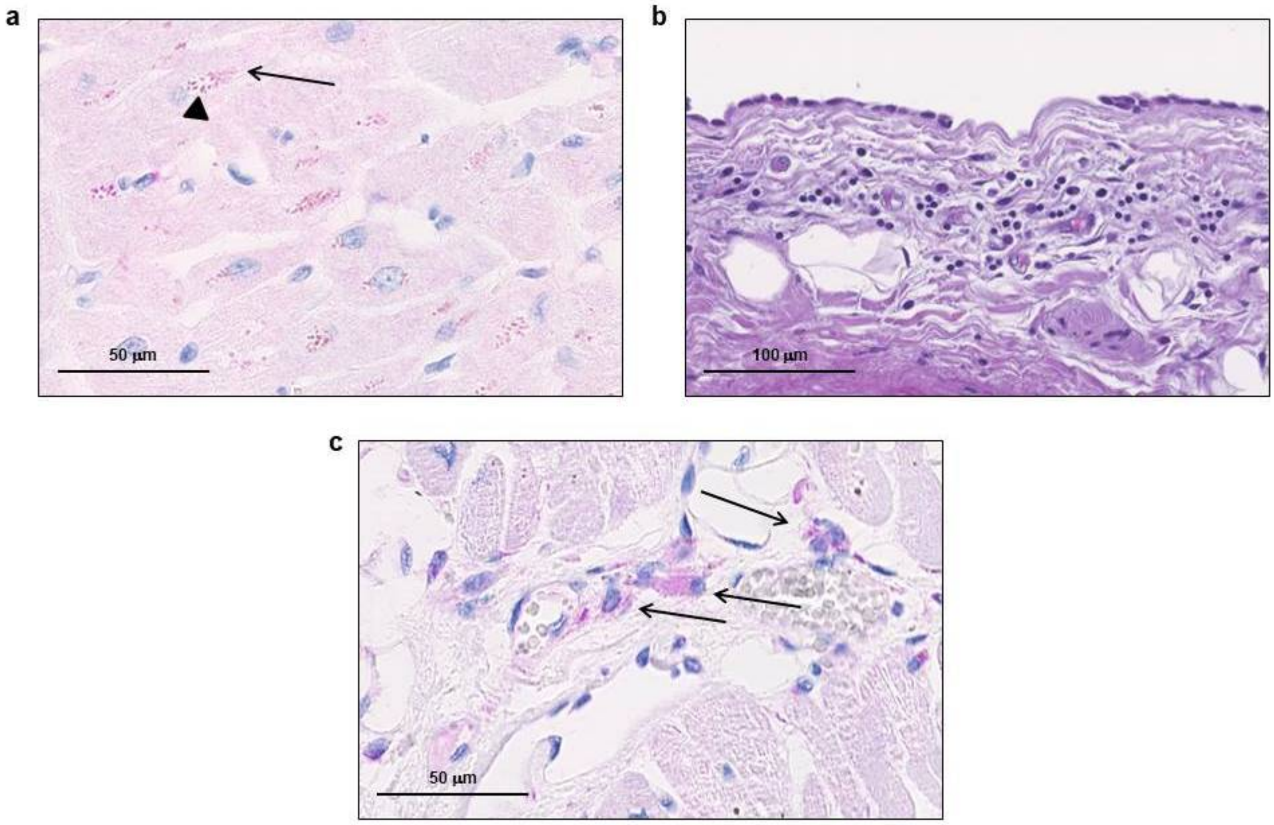

Supplementary Figure 4 – SARS-CoV-2 nucleoprotein localization in lipofuscin granule area of cardiomyocytes and in macrophages, and inflammatory infiltrates of heart samples of COVID-19 patients. a) Representative image of immunohistochemistry on 3µm-slides of formalin-fixed paraffin-embedded left ventricle specimens from COVID-19-positive patient for SARS-CoV-2 nucleoprotein. The lipofuscin granules (in light brown) are indicated with arrowhead, while viral nucleoprotein (in red), localized also in same sub-cellular area, is indicated with black arrow. **b)** Haematoxylin-eosin staining on left ventricle specimens from COVID-19-positive patient. The inflammatory infiltrate is clearly localized in sub-epicardial region. **c)** Representative image of immunohistochemistry on left ventricle specimens from COVID-19-positive patient for SARS-CoV-2 nucleoprotein (in red). The high immunoreactive interstitial macrophages are indicated with black arrows. Magnification as scale bars.

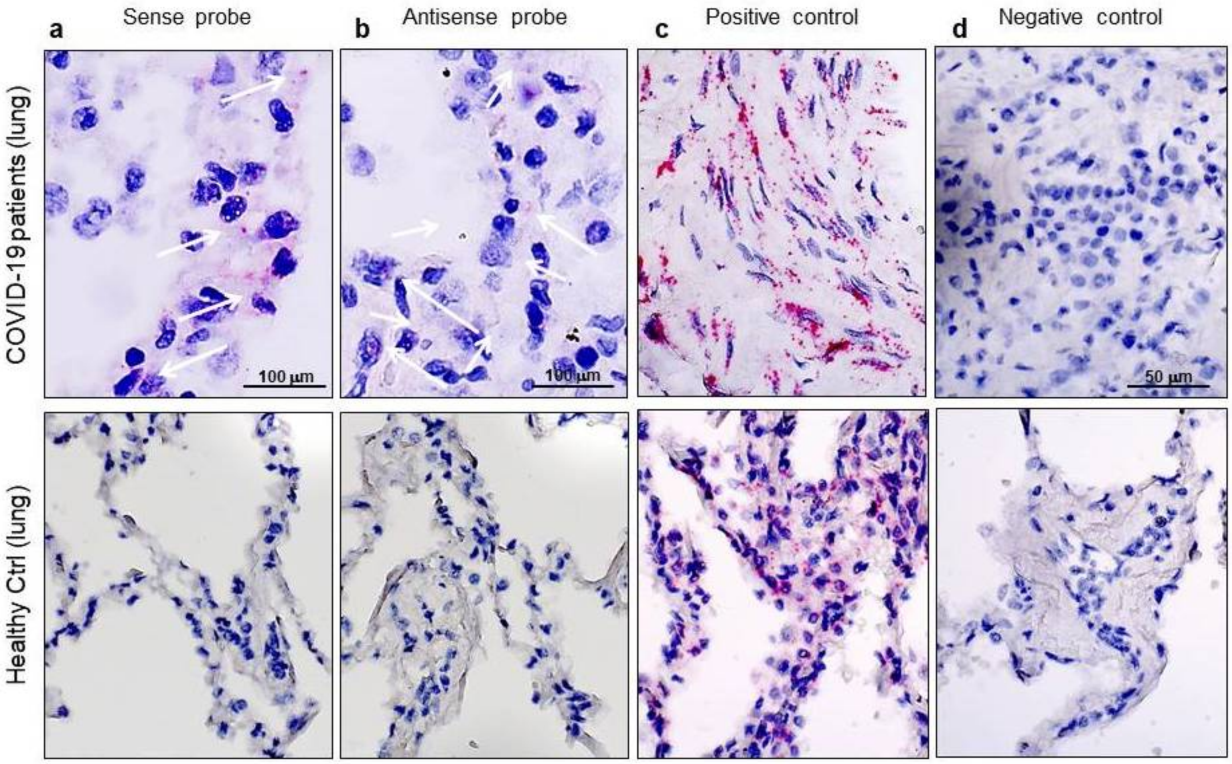

Supplementary Figure 5 - SARS-CoV-2 sense and antisense RNA are localized in the lung specimens of COVID-19 patients. a-b) Representative images of spatially resolved viral RNA detection by RNAScope assay on 3µm-slides of formalin-fixed paraffin-embedded lung specimens from COVID-19 patients (upper panels) and healthy control (Healthy Ctrl, lower panels) for SARS-CoV-2 sense (**a**) and antisense (**b**) probes for spike protein RNA sequences, as well as probes for human PPIB gene (**c**, positive control) and for bacterial DapB gene (**d**, negative control). Fast Red dots indicate viral RNA presence (white arrows). Nuclei have been counterstained with haematoxylin. Magnification as scale bars.

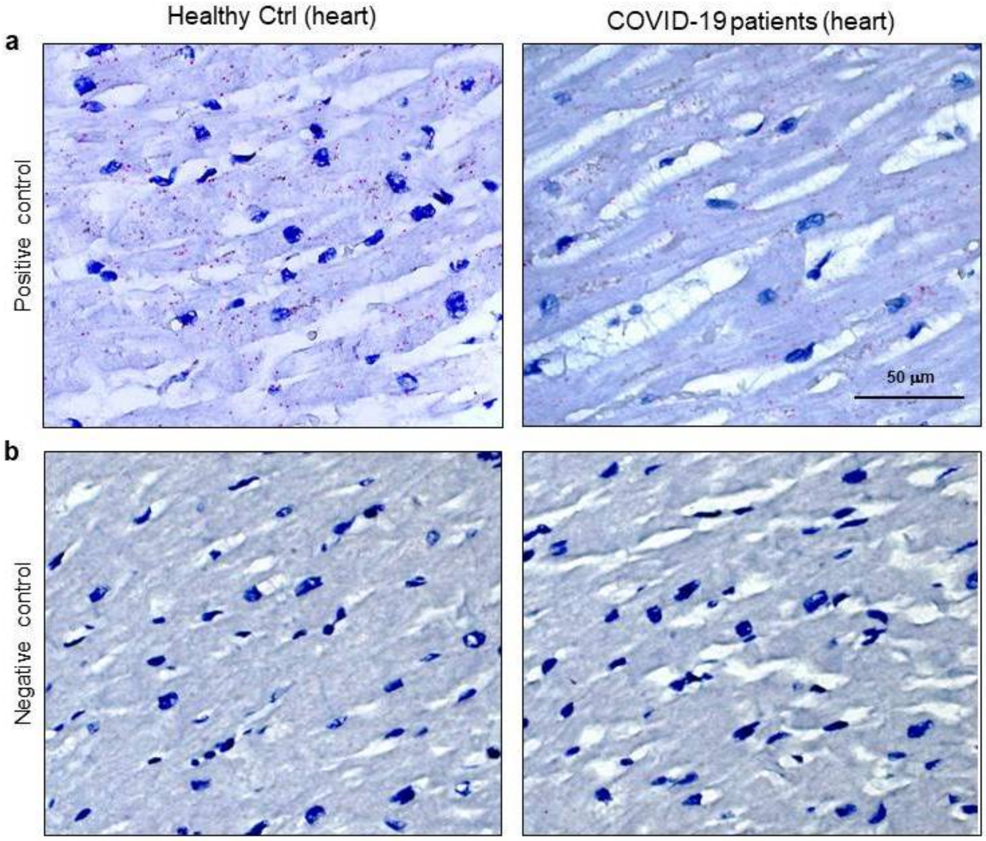

Supplementary Figure 6 – RNAScope positive and negative controls on heart samples from healthy subject and COVID-19 patients. a-b) Representative images of spatially resolved RNA detection by RNAScope assay on 3µm-slides of formalin-fixed paraffin-embedded left ventricle specimens from healthy control (Healthy Ctrl, left panels) and COVID-19-positive patients (right panels). The positive control probe targets human PPIB gene (**a**), while the negative control the bacterial DapB gene (**b**). Fast Red dots indicate RNA. Nuclei have been counterstained with haematoxylin. Magnification as scale bars.
